# Introductory

**Published:** 1908-11-28

**Authors:** 


### Motoring Notes.

### INTRODUCTORY.

At the present season, just after the Olympia Show, the thoughts of most medical men are turned towards the motor car. Many of those who have used the car are thinking of a new one or are interested in the latest models; others who hitherto have been weighing the pros and cons, and who in a measure have been "shivering on the brink," have decided to take the plunge and abandon the antiquated horsed vehicle for the much more up-to-date and very much more efficient modern motor car. To the latter class I would say, hesitate no longer; motor cars are not now, as they were a few years ago, in the experimental stage, when breakdowns were frequent and repair bills large. Pneumatic tyres, admittedly to-day the weakest point in the car, have been so improved in construction that, provided their size is proportionate to the weight and power of the car, they give very little trouble. In short, I would say that the motor car of to-day is one of the most reliable means of locomotion, and costs have now been so reduced that it is the cheapest of all private conveyances. The subject of the cost of running a car is of such importance to the medical man that I propose in future articles to devote some space to this subject; but at the present time I intend to confine myself to a short description of the various cars now on the market which are suitable for a doctor.

I may say that, with one or two exceptions, the manufacturers have practically neglected the small car. All their energies have been devoted to designing and manufacturing large powerful cars. This has resulted in over-production, and more or less of a crisis in the industry, with the inevitable consequence that manufacturers have at last realised that the field for disposal of the large powerful car is extremely small when compared with that of its lesser brother. Thus to-day small cars are being turned out by firms who, as recently as a year or so ago, would scarcely deign to recognise their existence. The increased number of small cars at Olympia is a proof of this, and I believe the Paris Salon, to be opened to-day, will contain next to no novelties in the way of big cars, while new types of small vehicles will be found on almost every stand. In fact, the Paris Show of 1908 will be known as "The Small Car Salon."

I now propose to give some particulars of cars that are suitable for medical men, both from the point of view of size and of price.

I think most medical motorists will agree with me that pride of place must be given to the De Dion-Bouton car. The De Dion-Bouton Company, whose works are, I believe, the largest in the world, unlike most big firms, have consistently catered for the medical motorist, and their small car with the famous expanding clutch gear, has a world-wide reputation for reliability and simplicity. This company specialises for the medical man, and publishes a booklet, "The Doctor's Motor Car," in which it is stated that over 500 medical men in this country are on their records as owners of their cars. For the coming season, since they are such firm believers in the future of the single-cylinder vehicle, they are supplying three varieties of the single-cylinder car. They point out that many motorists and writers refer to the single cylinder as obsolete, and speak as if nothing but four-cylinder cars were efficient; yet for anyone who wants a really high-class car at a low first cost, and designed for simplicity and economy, a first-class single-cylinder car is far preferable to a second-class multi-cylindered one. The three models are two 8 h.-p. cars and one 9 h.-p. One of the 8 h.-p. cars is practically the same car that they have been selling for the past six years. It has the expanding clutch gear, and of the 500 doctors on their register 80 per cent. drive this type. It is listed with two-seated body at £222. The other 8 h.-p. car will have the same engine, but is fitted with a sliding pinion type of gear and pedal-operated cone clutch. This is listed at £214 10s.

The 9 h.-p. car will have a plate clutch, automatic carburettor, forced lubrication, and high-tension magneto. The gear will be of the double-sliding type, vertical gate change, and direct drive on top speed. With two-seated body it is listed at £276. The chassis of this car will take a short side-entrance body. This firm has decided to discontinue manufacturing the two-cylinder car.

During the past few years the Rover Company, of Coventry, have built two cars which many medical men have favoured. I allude to the 6 and 8 h.-p. single-cylinder models. These are again to be supplied with various improvements in details. They both have accumulator ignition, single-plate clutch, and quadrant change speed gear. The price of the 6 h.-p. with two-seated body, hood, and screen is £150, that of the 8 h.-p. with two-seated body, but without hood and screen, is £210.

Messrs. Reynolds Jackson and Co. again show their well-known and easily recognised Jackson dog-cart. This car has been favoured by many motorists, and the type of body is one that certainly has many advantages. They also exhibit a model which they call the doctor's car. It is fitted with the De Dion-Bouton 8 h.p. single-cylinder engine, which Messrs. Jackson say they have selected on account of its reputation, which indisputably places it in the premier position of the automobile world. The frame is pressed steel, and the car has a long wheelbase, Renault type transmission with direct drive on high speed. The body is two-seated, nicely upholstered, and fitted with a leather hood and wind-screen, and is sold at £242. I may say that this firm's price includes such usual "extras" as side and tail lamps, horn, inflator, and tools.

This is a practice I should be pleased to see manufacturers more generally follow, as after paying for and receiving the car, it is annoying to find how many really necessary fitments the car requires, and still more annoying when one discovers the price to which these run.

